# Streptozotocin as a tool for induction of rat models of diabetes: a practical guide

**DOI:** 10.17179/excli2022-5720

**Published:** 2023-02-21

**Authors:** Asghar Ghasemi, Sajad Jeddi

**Affiliations:** 1 Endocrine Physiology Research Center, Research Institute for Endocrine Sciences, Shahid Beheshti University of Medical Sciences, Tehran, Iran

**Keywords:** animal model, rat, streptozotocin, diabetes

## Abstract

Streptozotocin (STZ) is the most used diabetogenic chemical for creating rat models of type 1 and type 2 diabetes. Despite ~60 years of using STZ in animal diabetes research, some prevailing views about STZ preparation and use are not supported by evidence. Here, we provide practical guides for using STZ to induce diabetes in rats. Susceptibility to the diabetogenic effect of STZ is inversely related to age, and males are more susceptible to STZ than females. Wistar and Sprague-Dawley rats, the most commonly-used rat strains, are sensitive to STZ, but some strains (e.g., Wistar-Kyoto rats) are less sensitive. STZ is mostly injected intravenously or intraperitoneally, but its intravenous injection produces more stable hyperglycemia. Despite the prevailing view, no fasting is necessary before STZ injection, and injection of its anomer-equilibrated solutions (i.e., more than 2 hours of dissolving) is recommended. Mortality following the injection of diabetogenic doses of STZ is due to severe hypoglycemia (during the first 24 h) or severe hyperglycemia (24 h after the injection and onwards). Some measures to prevent hypoglycemia-related mortality in rats include providing access to food soon after the injection, administration of glucose/sucrose solutions during the first 24-48 h after the injection, administration of STZ to fed animals, and using anomer-equilibrated solutions of STZ. Hyperglycemia-related mortality following injection of high doses of STZ can be overcome with insulin administration. In conclusion, STZ is a valuable chemical for inducing diabetes in rats, but some practical guides should be considered to perform well-conducted and ethical studies.

## Introduction

Diabetes, as the largest epidemic in human history (Zimmet, 2017[143]), is among the top 10 causes of adult death (Zimmet, 2017[143]; Zheng et al., 2018[142]), with a worldwide prevalence of ~10 % (IDF, 2019[51]). In addition, complications of diabetes impose much morbidity on affected individuals (Ghasemi and Norouzirad, 2019[37]). Thus, it is not surprising that a large amount of biomedical research is ongoing to offer a better understanding of the pathophysiology of diabetes to improve diabetes management. Because of human ethical considerations, animal models are extensively used in diabetes research for pharmacological testing, genetic studies, and understanding disease mechanisms (Srinivasan and Ramarao, 2007[124]; Franconi et al., 2008[27]; King, 2012[68]). A prerequisite for using animal models of diabetes is knowledge and familiarity with practical hints for creating the models. 

Streptozotocin (STZ) is the most prominent diabetogenic chemical (Ghasemi et al., 2014[36]) that is widely used in experimental animals for creating animal models of type 1 and type 2 diabetes (Samuel et al., 2014[115]). Obtaining valid data from STZ-based animal models of diabetes depends on the correct preparation and use of STZ. Despite the long history and extensive use of STZ in diabetes research, some essential points regarding STZ use (e.g., its preparation, suitable dose, and anomeric composition) are not always considered. These issues preclude appropriate comparison of results obtained by different studies and cause a loss-in-translation of animal data to humans. Suboptimal preclinical data in animal models is one cause of the limited success rate of drugs during clinical investigations (Singh and Seed, 2021[120]). In this review, we provide practical guides for using STZ in diabetes research that can assist researchers in conducting better studies.

## Animal Models of Diabetes

Models are needed when we can not put our hands on the object of the study (Wall and Shani, 2008[136]). An animal model is a living organism in which a phenomenon of interest, similar in some aspects to humans, is studied in a way that can not be studied in humans (Wall and Shani, 2008[136]). Using animal models in biomedical research has a long history (Wall and Shani, 2008[136]; Bahadoran et al., 2020[5]). An estimate in 2005 indicated that the number of laboratory animals used for research was about 115 million per year (Taylor et al., 2008[132]) and is still increasing (Goodman et al., 2015[40]; Hudson-Shore, 2016[49]). A report on toxicological studies indicates that the concordance between adverse findings in clinical data with data produced in experimental animals was 71 % (Olson et al., 2000[95]). In addition, according to both the Nuremberg Code (Shuster, 1997[119]) and the Declaration of Helsinki (Carlson et al., 2004[13]), which are cornerstones for conducting ethical biomedical research (Shuster, 1997[119]), animal studies need to be conducted before human trials. 

However, there is controversy concerning the predictive power of animal models (Shanks et al., 2009[118]). Results obtained from animals are not predictive of human response and should be only used for generating hypotheses to be tested in humans (Shanks et al., 2009[118]). A high rate of new drugs that passed preclinical studies (about nine out of ten (Shanks et al., 2009[118])) fail in the clinical phase (Singh and Seed, 2021[120]). For example, the average successful translation rate from animals to clinical cancer trials is < 8 % (Mak et al., 2014[80]). Thus, animal models are excellent basic science tools but are not appropriate for biomedical prediction. We can use the results of animal studies for building conceptual models to generate testable hypotheses to verify in humans (Wall and Shani, 2008[136]). In other words, animal models of human diseases provide an understanding of the studied disease and are not intended to act as a direct one-to-one surrogate (Wall and Shani, 2008[136]). 

Despite the limitations mentioned, animal models have remained the best alternative way for testing hypotheses before human trials (Wall and Shani, 2008[136]) and are a core of preclinical drug development, which is a lengthy and expensive process (Singh and Seed, 2021[120]). An ideal animal model should mimic natural disease patterns in humans as closely as possible (Islam and Loots du, 2009[53]); however, none of the models corresponds to human disease (Bell and Hye, 1983[9]; Franconi et al., 2008[27]), and each model provides advantages for studying some areas of the disease (Islam and Wilson, 2012[54]). 

Rats and mice are the animals of choice for diabetic studies (Islam and Loots du, 2009[53]), featured in 94 % of articles in the field of endocrinology (Beery and Zucker, 2011[8]); this is mainly because of easy availability and short generation interval (Srinivasan and Ramarao, 2007[124]). The rat model of diabetes is more similar to human disease, for example, in the ability of the agents to modify the disease (Iannaccone and Jacob, 2009[50]). Rats have a short gestation period (21-22 days) and reach sexual maturity at post-natal days 60-70 (Ghasemi et al., 2021[35]), making them the first choice of animal models in diabetes research (Islam and Choi, 2007[52]). Moreover, the larger size of rats compared to mice enables serial blood sampling more easily (Iannaccone and Jacob, 2009[50]).

Rodent models of diabetes can be categorized as genetic and experimentally induced. The latter has a lower cost and is simpler to induce and thus is widely used for research purposes (Islam and Loots du, 2009[53]; Ghasemi et al., 2014[36]). Experimentally-induced animal models of diabetes are produced by surgery, dietary handling, chemicals, or their combination (Islam and Wilson, 2012[54]; Ghasemi et al., 2014[36]). The most common types of diabetes are type 1 and type 2 diabetes that are associated with absolute and relative insulin deficiency, respectively (King, 2012[68]). Chemicals used for the induction of animal models of diabetes are STZ, alloxan, vactor, dithizone, 8-hydroxyquinolone, and gold thioglucose (Rees and Alcolado, 2005[106]; Tripathi and Verma, 2014[135]). 

STZ, the most prominent diabetogenic chemical (Ghasemi et al., 2014[36]), is widely used in experimental animals for creating animal models of type 1 and type 2 diabetes (Samuel et al., 2014[115]) (Table 1(Tab. 1); References in Table 1: Ar'Rajab and Ahrén, 1993[3]; Gajdosík et al., 1999[30]; Masiello et al., 1998[83]; Mythili et al., 2004[92]; Portha et al., 1974[101]; Reed et al., 2000[105]; Rossini et al., 1977[111]; Srinivasan et al., 2005[125]; Wang et al., 1996[137]; Weir et al., 1981[139]). In chemically-induced models of type 1 diabetes, both single high dose of STZ [35-65 mg/kg intravenously (IV) or intraperitoneally (IP)] (Srinivasan and Ramarao, 2007[124])) and multiple injections of sub-diabetogenic low dose of STZ (15 mg/kg for 5 consecutive days IV (Rossini et al., 1977[111]) or 20 mg/kg for 5 consecutive days IP (Lukić et al., 1998[76])) are used in rats. Among different models of experimentally-induced type 2 diabetes in rats (Ghasemi et al., 2014[36]), STZ is used in high-fat diet (HFD)/low doses of STZ (Reed et al., 2000[105]; Srinivasan et al., 2005[125]), STZ-nicotinamide (NA) (Masiello et al., 1998[83]), and neonatal STZ (Portha et al., 1974[101]) models.

In the initial study that introduced a combined fat-fed diet and STZ, Reed et al. used a moderate dose (50 mg/kg, IV) of STZ in male Sprague-Dawley rats after two weeks on HFD (40 % of total kcal from fat) (Reed et al., 2000[105]); in this model, 23 % of diabetic rats had serum glucose <250 mg/dL, 36 % had serum glucose 250-450 mg/dL, and 41 % had serum glucose >450 mg/dL (Reed et al., 2000[105]). Subsequently, a higher percentage of total kcal from fat (58 %) combined with a lower dose (35 mg/kg, IP) of STZ that was injected two weeks after HFD was used by Srinivasan et al. (2005[125]). In subsequent studies, STZ in the range of 15-40 mg/kg as well as percent of calories from fat in the range of 30-67 %, were used (Gheibi et al., 2017[38]). 

In the STZ-NA model, first introduced by Masiello et al., NA was injected 15 min before STZ injection to provide partial protection in β-cells; stable hyperglycemia, reduced pancreatic insulin content by 60 %, and insulin responsiveness to glucose are among the main advantages of this model; lack of insulin resistance is its main disadvantage (Masiello et al., 1998[83]). In subsequent studies that used this model, different doses of STZ (45-65 mg/kg, IV/IP) and NA (60-290 mg/kg, IP) was used 15-30 min before STZ injection (Ghasemi et al., 2014[36]).

In neonatal STZ rats, STZ (80-100 mg/kg (Srinivasan and Ramarao, 2007[124])) is injected on the day of birth (n0-STZ) (Portha et al., 1974[101]), two days after birth (Weir et al., 1981[139]) (n2-STZ), or five days after birth (n5-STZ) (Wang et al., 1996[137]). In neonatal STZ-treated rats, initial transient hyperglycemia is followed by normoglycemia, and non-fasting hyperglycemia is observed from 6-8 weeks of age (Masiello, 2006[82]). In addition, insulin secretion is insensitive to glucose, and abnormalities in the pancreas' α, β, and δ cells are observed (Weir et al., 1981[139]). When STZ is injected at more time after birth, the regenerating capacity of β-cells is lower; 3 weeks after STZ treatment, the number of β-cells in the pancreas was 23 % and 48 % lower in n2-STZ and n5-STZ, compared to n0-STZ rats (Wang et al., 1996[137]). Interested readers are referred to reviews on animal models of diabetes (Bell and Hye, 1983[9]; Rees and Alcolado, 2005[106]; Masiello, 2006[82]; Srinivasan and Ramarao, 2007[124]; Islam and Loots du, 2009[53]; King, 2012[68]; Ghasemi et al., 2014[36]; Tripathi and Verma, 2014[135]; Gheibi et al., 2017[38]; Kleinert et al., 2018[69]).

## Streptozotocin

Streptozotocin (also called Streptozocin) or 2-deoxy-2(([methyl(nitroso)amino]carbonyl)amino)-(α and β)-D-glucopyranose (Thurston and Pysz, 2021[134]), was discovered in 1959 as a natural antibiotic produced by *Streptomyces achromogenes*; its toxicity towards pancreatic β-cells (diabetogenic action) was reported in 1963 (Capdevila et al., 2022[12]) by Rakieten (Lenzen, 2008[74]). STZ molecule (molecular formula=C8H15N3O7, molecular weight≈265 (Junod et al., 1967[58])) has two parts: (1) glucopyranosyl group, which facilitates its uptake by pancreatic β-cells by glucose transporter 2 (GLUT2) and (2) nitrosourea group, which destructs pancreatic β-cells (Capdevila et al., 2022[12]). 

### STZ metabolism in rats

Studies in rats (Spanheimer, 1989[123]), mice (Anderson et al., 1974[2]), and humans (Kahn et al., 1975[60]) indicate that maximum plasma concentrations of STZ are lower than predicted values, indicating a very rapid initial metabolism of STZ in plasma. Immediately after injecting STZ (300 mg/kg, IV) in Sprague Dawley rats, serum STZ concentration was around 4 mM (Spanheimer, 1989[123]). The blood volume in rats can be estimated via the Lee and Blaufox formula

(Blood volume (mL) = 0.06 x Body weight (g) + 0.77)

(Lee and Blaufox, 1985[72]); in a 250 g rat, it would be 15.77 mL. Since hematocrit is about 40 % in a 3-months-old rat (Jacob Filho et al., 2018[56]), plasma volume would be ~9.5 mL. Therefore, STZ concentration in plasma after injection of 300 mg/kg of STZ is expected to be ~30 mM, 7.5 times the measured value. In male Swiss mice (body weight: 18-25 g), IP injection of STZ (100, 150, and 200 mg/kg, IP) provided maximum plasma STZ concentrations of 0.136, 0.161, and 0.224 mM (Anderson et al., 1974[2]). The blood volume in male Swiss mice can be calculated from the equation of Sluiter et al. 

(Blood volume (mL) = 0.07146 x Body weight (g))

(Sluiter et al., 1984[121]); thus, there is 1.3-1.8 mL blood in 18-25 g mice; since hematocrit is about 38 % in Swiss mice (Santos et al., 2016[116]), plasma volume would be 0.8-1.1 mL. Thus, expected concentrations of plasma STZ would be 8.4, 12.6,, and 16.9 mM following injection of 100, 150, and 200 mg/kg STZ, values that are 62, 78, and 75 times higher than the measured values. In a case study, STZ (200 mg) was administrated intravenously to a woman to treat pancreatic cholera, and the maximum plasma STZ concentration was 0.08 mM (Kahn et al., 1975[60]). Since plasma volume is about 2.5 L in women (Snyder et al., 1975[122]), plasma STZ concentration would be expected to be 0.3 mM, which is about 4 times higher than the measured value. 

After injection (70 mg/kg, IV) in rats, the plasma concentration of STZ is very high during the first 1-3 min and declines to relatively low levels in 15 min (Karunanayake et al., 1974[64]). Plasma half-life (time required that a given drug concentration decreases to one-half of its value (MacLeod et al., 1974[77])) of IV-injected STZ (60 mg/kg) is 5-6 min (Spanheimer, 1989[123]) or 6.9 min (Evan et al., 1984[23]) in Sprague-Dawley rats. STZ is rapidly cleared from the kidney in its unchanged form (Karunanayake et al., 1974[64]) or degraded in rat liver to produce excreted metabolites in the urine (Karunanayake et al., 1976[63]). Following IV injection of STZ (70 mg/kg) to male Sprague-Dawley rats, about 70 % of STZ appeared in the urine during 6 hours, with about 40 % exerted in the first hour after injection and peak urinary excretion observed at 10-20 min after injection (Karunanayake et al., 1976[63]). Only about 2-3 % of injected STZ excreted through bile over 6 hours (Karunanayake et al., 1976[63]). STZ is rapidly removed by the kidney and sealing the hilum of one kidney in rats for 5 min, during and following STZ injection (60 mg/kg, IV), prolongs its plasma half-life by about 70 % (from 6.9 to 11.7 min) (Evan et al., 1984[23]). 48 h after IV injection of ^[14C]^STZ (70 mg/kg) to Sprague-Dawley rats in both sex, about 80 % of STZ is excreted in the urine, 10 % in the feces and 10 % remains in the body, indicating rapid and extensive renal clearance of STZ (Karunanayake et al., 1974[64]). In addition to blood, STZ is distributed in the kidney, liver, and intestine (Karunanayake et al., 1974[64]), in which GLUT2 functions as major glucose transporter (Schnedl et al., 1994[117]).

### STZ toxicity in pancreatic β-cells

STZ has specific, rapid, and irreversible cytotoxic actions on pancreatic β-cells (Junod et al., 1967[58]). Destructive effects of STZ on β-cells start after 10 min of its IV injection; this notion is supported by experiments indicating that IP injection of NA together with or 10 min after STZ injection almost completely protects the pancreas of male Wistar rats against destructive effects of STZ (Stauffacher et al., 1970[126]). In addition, in an *in vitro* study, it has been shown that more than 60 % of STZ is degraded in plasma obtained from female Sprague-Dawley rats within 10 min and completely degraded in 4 hours (Lee et al., 1993[73]). 

In rodent β-cells, GLUT2 is the predominant glucose transporter (Berger and Zdzieblo, 2020[10]). In support of this hypothesis that the cytotoxic effect of STZ is associated with glucose transport capacity, RIN (rat insulinoma cell line) cells, which do not express GLUT2 and express GLUT1 instead, show resistance against STZ toxicity (65 % vs. 87 % and 49 % vs. 81 % cell viability in the presence of 10 mM and 20 mM of STZ for GLUT2-expressing and untransfected RIN cells, respectively) (Schnedl et al., 1994[117]). Overexpressing GLUT2 in RIN cells causes STZ transportation with high affinity and, thus, β-cell toxicity (Schnedl et al., 1994[117]; Elsner et al., 2000[22]). In addition, human islets, in which GLUT3, and in particular, GLUT1, play the major role in glucose transport, are resistant to STZ (Berger and Zdzieblo, 2020[10]). Moreover, K_ATP_ channels deficient mice are resistant to the diabetogenic effect of STZ because of lower GLUT2 activity (Xu et al., 2008[141]). STZ causes pancreatic β-cell death (apoptosis and necrosis) via different mechanisms, including DNA alkylation, depletion of cellular NAD^+^ levels and thus energy deprivation, increasing oxidative stress, and increasing nitric oxide production (Ghasemi et al., 2014[36]). 

### Glycemic response to STZ

When diabetogenic doses of STZ (45-65 mg/kg) is injected to male white Wistar rats, a triphasic response is observed in blood glucose concentration (Junod et al., 1967[58], 1969[59]; Gajdosík et al., 1999[30]): (1) early transient hyperglycemia (2-4 h after STZ injection) probably due to adrenaline response and sudden breakdown of liver glycogen without a parallel increase in serum insulin; (2) transient hypoglycemia (7-10 h after STZ injection) due to increased serum insulin because of insulin release from necrotizing β-cells but without a decrease in pancreatic insulin content; (3) stable hyperglycemia (24 h after STZ injection and onwards); in this phase, frank diabetes characterized by fasting permanent hyperglycemia, relative hypoinsulinemia (i.e., serum insulin was comparable with fasted normal animals but low related to coexisting hyperglycemia), polyuria, glycosuria, and marked (>90 %) decrease in pancreatic insulin content (Junod et al., 1969[59]). 

### Dose-dependent effects of STZ on circulation glucose

**Figure 1:**
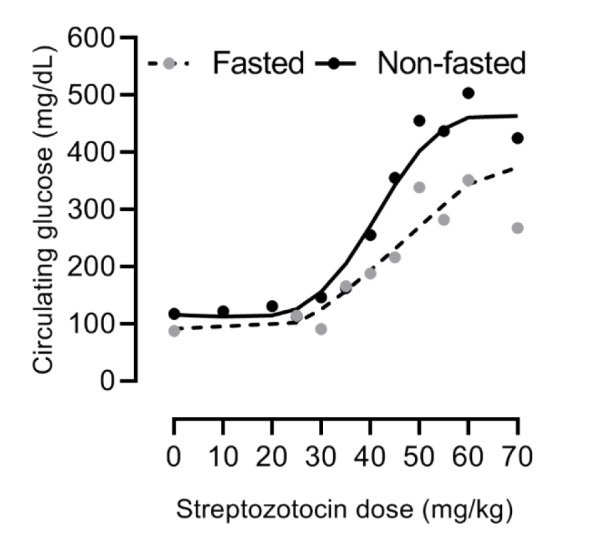
Relation between streptozotocin dose and fasted (14-16 hours) and non-fasted circulating glucose, recorded from 24 h to 4 months after the streptozotocin injection in rats. Data were obtained from (Junod et al., 1969; Babu and Srinivasan, 1997; Rodrigues et al., 1997; Mythili et al., 2004) and (Hoftiezer and Carpenter, 1973; Bar-On et al., 1976; Ganda et al., 1976; Evan et al., 1984; Ar'Rajab and Ahrén, 1993; Rodrigues et al., 1997; Masiello et al., 1998; Gajdosík et al., 1999; Srinivasan et al., 2005; Akbarzadeh et al., 2007) for fasted and non-fasted states, respectively. In the case that data was presented in a graph, values were extracted using Photoshop, as previously reported (Gheibi et al., 2019).

There is an association between the dose of STZ injected and the diabetic state induced (Tancrède et al., 1983[129]). As shown in Figure 1(Fig. 1) (References in Figure 1: Akbarzadeh et al., 2007[1]; Ar'Rajab and Ahrén, 1993[3]; Babu and Srinivasan, 1997[4]; Bar-On et al., 1976[6]; Evan et al., 1984[23]; Gajdosík et al., 1999[30]; Ganda et al., 1976[31]; Gheibi et al., 2019[39]; Hoftiezer and Carpenter, 1973[46]; Junod et al., 1969[59]; Masiello et al., 1998[83]; Mythili et al., 2004[92]; Rodrigues et al., 1997[110]; Srinivasan et al., 2005[125]), the association between serum glucose and the dose of STZ in rats has a sigmoidal shape and is quite steep in the range of 25-55 mg/kg (Junod et al., 1969[59]). Doses of 10 mg/kg (Ganda et al., 1976[31]), 20 mg/kg (Junod et al., 1969[59]; Ganda et al., 1976[31]), and 25 mg/kg (Junod et al., 1969[59]; Bar-On et al., 1976[6]) of STZ have no hyperglycemic effect in male rats (Ganda et al., 1976[31]). Doses of 30 mg/kg and greater create progressive hyperglycemia, with plasma glucose levels reaching a plateau at 60 mg/kg when measured 48 h after IV injection (Ganda et al., 1976[31]); there was no difference between fasting plasma glucose at doses of 60, 80, and 120 mg/kg in male rats (Ganda et al., 1976[31]). Doses of 30-40 mg/kg of STZ produce transient diabetes with spontaneous recovery, but doses of 50-70 mg/kg produce long-lasting diabetes (Ar'Rajab and Ahrén, 1993[3]) associated with severe hyperglycemia and major clinical signs of diabetes (Gajdosík et al., 1999[30]). Although doses of 35-65 mg/kg of STZ (IP or IV) are used for inducing type 1 diabetes in rats (Islam and Loots du, 2009[53]), 60 mg/kg has been suggested as the commonly used diabetogenic dose of STZ in rodents (Samuel et al., 2014[115]). However, it has been reported that a 40 mg/kg dose of IP STZ is optimal for creating diabetes with moderate hyperglycemia in Wistar (male and female) rats (Mythili et al., 2004[92]). Overall, STZ doses < 35 mg/kg, 40-55, and ≥ 60 mg/ kg are considered as low (sub-diabetogenic), intermediate (Islam and Wilson, 2012[54]), and high doses (Islam and Wilson, 2012[54]), respectively (Rodrigues et al., 1997[110]; Islam and Wilson, 2012[54]). LD_50_ of STZ in rats is about 130 mg/kg (Junod et al., 1967[58]), which is lower than that in mice (345 mg/kg (Levine et al., 1980[75])). The dose of STZ should be optimized so that diabetes is successfully induced and, simultaneously, significant mortality is avoided (Goyal et al., 2016[41]). Factors that should be considered when a dose of STZ is used include animal age (Masiello et al., 1975[84], 1979[85]), sex (Rossini et al., 1978[113]; Tesch and Allen, 2007[133]; Kim et al., 2020[67]; Saadane et al., 2020[114]), strain (Rodrigues et al., 1997[110]; Hayashi et al., 2006[45]), and the route of administration (Tesch and Allen, 2007[133]). Therefore, it is suggested that for producing permanent stable hyperglycemia, researchers optimize the dose of STZ between 45-65 mg/kg, considering the above-mentioned factors.

The other point inferred from Figure 1(Fig. 1) is that following STZ injection, circulation glucose is different in fasted and non-fasted animals. The difference between fasted and non-fasted circulating glucose is more pronounced in intermediated doses (e.g., 55 mg/kg, IV) in male Wistar rats (249±36 vs. 533±13 mg/dL, 114 % difference). This difference is less in control rats (122±5.4 vs. 135±1.8 mg/dL, 11 % difference) and those that received a high dose (75 mg/kg, IV) of STZ (485±14 vs. 542±18 mg/dL, 12 % difference) (Rodrigues et al., 1997[110]). Fasting before the blood sampling in rodents is recommended to reduce the variation in blood glucose readings associated with feeding habits. It is recommended that blood glucose be measured in fasted animals (Tesch and Allen, 2007[133]), including rats (Goyal et al., 2016[41]). However, it should be decided case by case (Matsuzawa and Sakazume, 1994[86]) based on study objectives (Weingand et al., 1996[138]). Duration of fasting is an important factor that can cause variations in data during experimental studies (Kale et al., 2009[62]). A 16 h fasting duration has been recommended for preclinical studies involving clinical pathology measurement in rats (Kale et al., 2009[62]).

### Dose-dependent effects of STZ on pancreatic insulin contents

**Figure 2:**
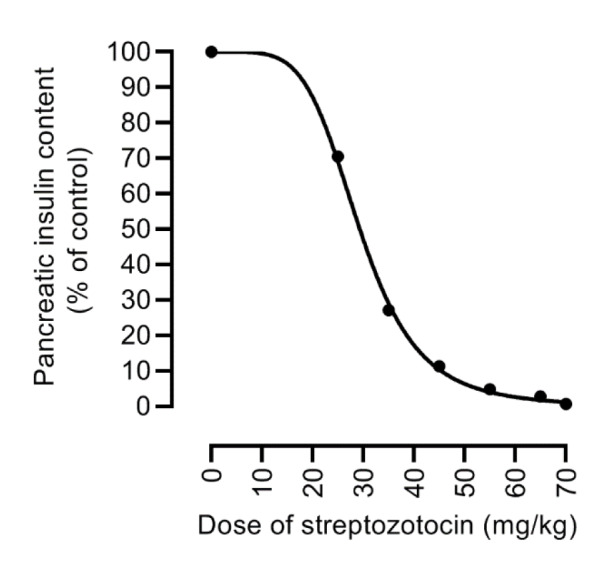
Relation between streptozotocin dose and pancreatic insulin content, recorded from 24 h to 35 days after the streptozotocin injection in rats. Data were obtained from (Junod et al., 1967, 1969; Stauffacher et al., 1970; Robbins et al., 1980; Rodrigues et al., 1997). In the case that data was presented in a graph, values were extracted using Photoshop, as previously reported (Gheibi et al., 2019).

It has been suggested that the best index of the diabetogenic activity of STZ is the pancreatic insulin content 24 h after its IV injection (Junod et al., 1969[59]). As shown in Figure 2(Fig. 2) (References in Figure 2: Gheibi et al., 2019[39]; Junod et al., 1967[58], 1969[59]; Robbins et al., 1980[109]; Rodrigues et al., 1997[110]; Stauffacher et al., 1970[126]), pancreatic insulin content is progressively decreased with increasing doses of the injected STZ. STZ (20 mg/kg) did not affect pancreatic insulin content seven days after IV injection (Junod et al., 1969[59]). At the dose of 25 mg/kg of STZ, the only change observed was a decrease in pancreatic insulin content by 36 % and 25 % following 24 h and 7 days of STZ injection (Junod et al., 1969[59]). Pancreatic insulin contents were decreased by 84 % and 61 % 24 h after 65 and 55 mg/kg STZ injection (Junod et al., 1969[59]). It seems that fasting hyperglycemia becomes evident following doses of STZ that deplete pancreatic insulin content by 65-75 % (≥ 35 mg/kg) (Junod et al., 1969[59]). Humans maintain normoglycemia until 50 % of the β-cells mass is lost (Meier, 2008[89]). Decreased β-cell mass in long-lasting type 1 diabetes is about 98 % (Meier et al., 2005[90]) due to immune-mediated β-cell destruction (Meier, 2008[89]). Mostly due to apoptosis-induced decreased number of β-cells, β-cell mass is 63 % lower in obese humans with type 2 diabetes compared to obese nondiabetic subjects; this value was 41 % lower in lean subjects with type 2 diabetes compared to lean nondiabetic subjects (Butler et al., 2003[11]). 

## Is Fasting before STZ Injection Advantageous?

Before the injection of STZ in rats, different fasting times have been used in studies, including 6 h (Cruz et al., 2021[17]), 12 h (Mostafavinia et al., 2016[91]), 16 h (Junod et al., 1969[59]), 20 h (Katada and Ui, 1977[65]), 24 h (Hoftiezer and Carpenter, 1973[46]; Ganda et al., 1976[31]; Rossini et al., 1977[112]), and 72 h (Chi et al., 2007[15]). Some authors also vaguely reported that STZ was injected after overnight fasting (Gajdosík et al., 1999[30]; Su et al., 2006[127]). What is the rationale behind the injection of STZ to fasted animals? The answer is to minimize the competition between glucose and STZ for GLUT2-mediated uptake into the β-cell (King, 2012[68]; Chaudhry et al., 2013[14]). Nevertheless, is this answer supported by evidence? 

Glucose uptake in rat β-cells is mainly mediated through GLUT2 (Berger and Zdzieblo, 2020[10]), which has a very high V_max_ (32 mmol/min/L islet space) and a high K_m_ (17 mM) for glucose (Johnson et al., 1990[57]). The high transport capacity of GLUT2 causes rapid equilibrium between extracellular and intracellular glucose (Berger and Zdzieblo, 2020[10]). In contrast, K_m_ of glucokinase (4-10 mM), which phosphorylates glucose inside the β-cell is much lower than GLUT2; in fact, glucose uptake in rat β-cells is about 66 and 88 times faster than glucose utilization by glucokinase (Berger and Zdzieblo, 2020[10]). These data indicate that glucokinase-dependent glucose phosphorylation is the rate-limiting factor for glucose utilization in β-cell rather than GLUT2-mediated glucose uptake (Berger and Zdzieblo, 2020[10]). In addition, infusion of glucose (13.75 mmol/kg) to male rats had no protective effect against STZ (60 mg/kg, IV), and plasma glucose concentrations were comparable between saline-infused and glucose-infused rats (422±7.5 vs. 408±27.9 mg/dL) 48 h later (Ganda et al., 1976[31]). On the other hand, 3-O-methyl glucose (6.39 mmol/kg) and 2-deoxyglucose (7.5 mmol/kg), which are nonmetabolized analogs of glucose, provided protection against STZ (60 mg/kg)-induced hyperglycemia by about 70 % and 40 %, respectively (Ganda et al., 1976[31]), indicating that β-cell glucose metabolism, not GLUT2-mediated uptake, is the limiting factor for STZ efficacy (Chaudhry et al., 2013[14]). In further support, following the injection of a diabetogenic dose of STZ (70 mg/kg, IV) in rats, small amounts lower than that found in the blood reach the pancreas (Karunanayake et al., 1974[64]). Plasma glucose concentrations vary between a minimum of 55 mg/dL (3.1 mM) during fasting to a maximum of 160 mg/dL (8.9 mM) after a meal, and its daily average is about 90 mg/dL (5.0 mM) (Rizza et al., 1980[108]; Gerich, 1993[33]; Defronzo, 2009[20]; Gerich, 2010[34]). Considering Michaelis-Menten equation


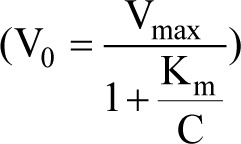
,

where V_0_, is the initial transport rate; V_max_, is maximum rate of transport, K_m_, is glucose concentration at which V_0_ equals to V_max _/ 2, and C, is the extracellular glucose concentration, rates of glucose transport by GLUT2 are 15 %, 23 %, and 34 % at glucose concentrations of 3.1, 5.0, and 8.9 mM. These values indicate that even at the postprandial state, GLUT2 works with about one-third of its capacity for transporting glucose and that competition between STZ and glucose for uptake by β-cells has little importance. 

Results of a study in male mice indicate that injecting multiple low doses of STZ was equally effective for inducing hyperglycemia, glucose intolerance, β-cell dysfunction, and β-cell loss in fed and fasted (6 h: 10:00-16:00) mice and therefore recommends that fasting before STZ administration is not required (Chaudhry et al., 2013[14]). Another report in male and female mice indicates that injecting a single dose of STZ in non-fasted animals can successfully induce diabetes (de la Garza-Rodea et al., 2010[18]). There are also reports of injecting STZ in non-fasting male Wistar (Tancrède et al., 1983[129]) and Sprague-Dawley (Ar'Rajab and Ahrén, 1993[3]) rats with successful induction of hyperglycemia.

Injection of STZ in non-fasted rats prevents metabolic stress and weight loss (Chaudhry et al., 2013[14]). Fasting decreases rat body weight by about 5 % (Matsuzawa and Sakazume, 1994[86]) and 10 % (Maejima and Nagase, 1991[79]) following 16 h and 24 h, respectively. Fasting causes a time-dependent decrease in body weight in both male (0.25-0.48 %/h over 4-48 h) and female (0.22-0.50 %/h over 4-48 h) Wistar rats (Kale et al., 2009[62]). Fasting also alters serum glucose in normal (Kale et al., 2009[62]) and diabetic (Islam and Choi, 2007[52]) rats. 16 h, 24 h, and 48 h of fasting caused 42.2 %, 43.7 %, and 51.1 % decrease in serum glucose concentrations in normal male Wistar rats and 27.6 %, 35.2 %, and 41.4 % in normal female Wistar rats (Kale et al., 2009[62]). Shorter fasting periods (4 and 8 hours) did not affect serum glucose concentrations in rats (Kale et al., 2009[62]), which indicates their wastefulness before STZ injection, unlike recommendations by some papers (Goyal et al., 2016[41]). In male Sprague-Dawley rats treated with HFD and STZ (30 mg/kg, IP), non-fasting blood glucose, as measured one week after STZ injection, was 317 % higher than fasting glucose, measured 48-72 h after STZ injection (371 vs. 89 mg/dL); in addition, fasting glucose was in normal range whereas non-fasting glucose was in the diabetic range (Islam and Choi, 2007[52]).

Overall, a short answer to the question posed in the heading is that fasting before STZ injection to induce diabetes is unnecessary and not recommended in mice (de la Garza-Rodea et al., 2010[18]; Chaudhry et al., 2013[14]) and rats (Goyal et al., 2016[41]).

## Preparation of STZ Solution

STZ is a pale yellow, freeze-dried powder (Frost et al., 2015[28]) stored at -20 ºC (de la Garza-Rodea et al., 2010[18]). STZ is a hydrophilic agent (Lenzen, 2008[74]) and is found in two anomeric forms of α and β (de la Garza-Rodea et al., 2010[18]). Based on the first reports on the diabetogenic action of STZ, STZ solution is prepared and maintained in the ice-cold acidic (pH 4.5) citrate buffer and is used immediately after its preparation (Junod et al., 1967[58], 1969[59]; Ganda et al., 1976[31]; Mythili et al., 2004[92]; Tesch and Allen, 2007[133]). It has been mentioned that STZ solution is stable only at a pH of 4.2 to 4.5 (Mythili et al., 2004[92]) and is not stable in an unbuffered aqueous solution (Oles, 1978[94]). Therefore, freshly prepared and immediate injection of STZ for inducing diabetes has become routine among most researchers (de la Garza-Rodea et al., 2010[18]; King, 2012[68]). Most researchers use acidic (pH 4.0-4.7) citrate-buffered solutions to prepare STZ for injection (Bar-On et al., 1976[6]; Karunanayake et al., 1976[63]; Rossini et al., 1977[112]; Robbins et al., 1980[109]; Tancrède et al., 1983[129]; Ar'Rajab and Ahrén, 1993[3]; Babu and Srinivasan, 1997[4]; Gajdosík et al., 1999[30]; Mythili et al., 2004[92]; L'Abbate et al., 2007[70]; Tesch and Allen, 2007[133]; Lebed et al., 2008[71]; Palsamy and Subramanian, 2011[97]; Ramzy et al., 2014[103]). Others use saline (Evan et al., 1984[23]), acidified (pH, 4.3-4.5) saline (Junod et al., 1967[58], 1969[59]; Stauffacher et al., 1970[126]; Hoftiezer and Carpenter, 1973[46]), and rarely phosphate buffer saline (pH, 7.4) (Chaudhry et al., 2013[14]), or saline (pH 7.2) (Rodrigues et al., 1997[110]) to prepare STZ for injection. The concentration of citrate buffer, which is the most used solvent, varies 10000-fold and concentrations of 0.01 mM (Ramzy et al., 2014[103]), 5 mM (Anderson et al., 1974[2]; Portha et al., 1974[101]), 10 mM (Karunanayake et al., 1976[63]; Tesch and Allen, 2007[133]), 50 mM (Bar-On et al., 1976[6]; Schnedl et al., 1994[117]; Kim et al., 2020[67]), and 100 mM (Ganda et al., 1976[31]; Robbins et al., 1980[109]; Ar'Rajab and Ahrén, 1993[3]; Babu and Srinivasan, 1997[4]; Gajdosík et al., 1999[30]; Lebed et al., 2008[71]; Palsamy and Subramanian, 2011[97]) have been used. Some researchers emphasized that they prepared the solution immediately before injection (Junod et al., 1969[59]; Karunanayake et al., 1974[64], 1976[63]; Tancrède et al., 1983[129]; Rodrigues et al., 1997[110]; Gajdosík et al., 1999[30]; L'Abbate et al., 2007[70]), and others highlighted that they prepared STZ solution within 5 min (Bar-On et al., 1976[6]; Chaudhry et al., 2013[14]; Ramzy et al., 2014[103]), 10 min (Ganda et al., 1976[31]), or even 30-45 sec (Rossini et al., 1977[112]) before the injection. 

Based on the prevailing notion that dissolved STZ is quickly degraded, there are some recommendations in the literature, including (a) STZ is stable only at a pH of 4.2 to 4.5 (Mythili et al., 2004[92]), and to minimize decomposition of the drug, it needs to be prepared in ice-cold citrate buffer and maintained on ice (Mythili et al., 2004[92]); (b) citrate buffer used for STZ preparation should be fresh or frozen as aliquots at -20 ºC (Tesch and Allen, 2007[133]). Therefore STZ is usually administered in NaCl or citrate buffer solutions with acidic pH (4.0 to 4.8) (Lee et al., 1993[73]). Does evidence support these recommendations? 

It has been reported that STZ in citrate buffer (100 mM, pH 4) is stable for 4 h at room temperature with about 4 % degradation (Spanheimer, 1989[123]). After dissolving STZ in 100 mM citrate buffer, pH=4.5, kept in the dark at 4 ºC, its rate of degradation was 0.1 % daily as measured for 27 months; when kept at room temperature, its rate of degradation was 1 % daily as measured over 5 days of storage (de la Garza-Rodea et al., 2010[18]). STZ degradation, prepared in citrate buffered saline (pH 4.4) kept in the dark at 37 ºC, was 20 % during 14 days (about 1.4 % daily) (Premilovac et al., 2017[102]). However, another study indicates that STZ solution prepared in PBS buffer (pH 7.4) and kept at 37 ºC is almost completely degraded in 4 hours (Lee et al., 1993[73]). This data suggest that the essential factors for maintaining the stability of STZ are probably acidic citrate buffer, protection against light, and low temperature. This suggestion is partly in line with previous recommendations that STZ gives the most stable solution when dissolved in acidic (pH 4.5) citrate buffer (Lenzen, 2008[74]) and should be maintained in aluminum foil-wrapped tubes because it is light-sensitive (Tesch and Allen, 2007[133]; Furman, 2021[29]). However, an acidic solution can cause red blood cell hemolysis in rats and provide pain and discomfort to the animal (Lee et al., 1993[73]). STZ has been used for treating pancreatic neuroendocrine tumors since 1982 after the approval of the FDA (US Food and Drug Administration) (Capdevila et al., 2022[12]). In addition to 1 g of STZ, each vial of STZ powder contains 200-220 mg citric acid (Akbarzadeh et al., 2007[1]; El-Rashedy et al., 2013[21]; Frost et al., 2015[28]), providing a pH of 3.5-4.5 after dissolving in 9.5 mL of cold 0.9 % NaCl (Akbarzadeh et al., 2007[1]; Frost et al., 2015[28]). STZ solutions are maintained at 2-8 ºC and away from light (Akbarzadeh et al., 2007[1]; El-Rashedy et al., 2013[21]; Frost et al., 2015[28]).

STZ is found in two anomeric forms of α and β (de la Garza-Rodea et al., 2010[18]). The proportion of α anomer varies between different STZ lots (de la Garza-Rodea et al., 2010[18]). For example, the α-STZ percentage in lots prepared by the Upjohn Company (1970 to 1975) ranged from 25 % to 90 %, which can act as a source of variation (Rossini et al., 1977[112]). α-anomer is further toxic compared to β-anomer against pancreatic β-cells (de la Garza-Rodea et al., 2010[18]). Injecting α-STZ (90 % α-anomer and 10 % β-anomer) at doses of 30, 35, 40, and 45 mg/kg IV to fasted (24 h) male Sprague-Dawley rats caused higher blood glucose levels 48 h after the injection compared to injecting β-STZ (25 % α-anomer and 75 % β-anomer) (Rossini et al., 1977[112]). Still, it did not occur for lower doses (10 and 20 mg/kg) and higher doses (50, 55, and 60 mg/kg) (Rossini et al., 1977[112]). These data indicate that α-anomer is more diabetogenic at doses of 30, 35, 40, and 45 mg/kg (Rossini et al., 1977[112]). 

During the first 15 min after preparing different lots of STZ (84-88 % α-anomer) solution, α-anomer is 3-20-folds higher in the solution than β anomer (de la Garza-Rodea et al., 2010[18]). With time, α-anomer decreases and β-anomer increases, and equilibrium between anomers (50:50) is achieved during 60 min (de la Garza-Rodea et al., 2010[18]) or 60-90 min (Oles, 1978[94]) after dissolution. After 2 h and onwards, a ratio of 44 % α and 56 % β is found in the STZ solution (de la Garza-Rodea et al., 2010[18]). The rate of attainment equilibrium in the mutarotation reaction is pH-dependent and is slightly faster in lower pH (Oles, 1978[94]). Following IP STZ injection in non-fasted mice, blood glucose was 20 % higher in mice that received freshly-dissolved STZ, which mainly contains α-anomer, compared to those that received anomer-equilibrated solutions (de la Garza-Rodea et al., 2010[18]).

Despite the prevailing view that STZ should be injected freshly and immediately after dissolution, it has been recommended that the induction of diabetes in animals should be done by anomer-equilibrated solutions (de la Garza-Rodea et al., 2010[18]). This practice allows more reliable comparison of results from different laboratories by eliminating the effect of STZ anomer (de la Garza-Rodea et al., 2010[18]) and can help to provide more reproducible results when various lots of STZ are used (Rossini et al., 1977[112]). In addition, the relative amounts of the STZ anomers in a given preparation must be reported in studies of STZ-induced β-cell necrosis (Rossini et al., 1977[112]), an issue that is not the case in most studies. In support of injecting anomer-equilibrated solutions of STZ for creating an animal model of diabetes, the rate of reported mortality following IP STZ injection to non-fated mice was significantly lower after injection of the anomer-equilibrated solution (stored solution of STZ in 0.1 M citrate buffer, pH=4.5, kept in the dark at 4 ºC and injected 2-3 hours after dissolution) compared to freshly-prepared (injected within 15 min after dissolution) solution (7 % vs. 36 %) (de la Garza-Rodea et al., 2010[18]). 

To sum up, injecting anomer-equilibrated solutions of STZ (i.e., more than 2 hours of dissolving) prepared in acidic (pH 4.5) citrate buffer solution and kept in the dark (aluminum foil-wrapped tubes) is recommended for inducing diabetes in laboratory animals.

## Sex Differences in Response to STZ

Glucose homeostasis (Mauvais-Jarvis, 2018[87]), β-cell function (Gannon et al., 2018[32]), insulin sensitivity (Mauvais-Jarvis, 2018[87]), and type 2 diabetes (Franconi et al., 2008[27]) are sex-dependent phenomena. Women have about 6 % more β-cells than men (Marchese et al., 2015[81]), and more severe adverse health consequences of diabetes occur in women than men (Franconi et al., 2008[27]). Impaired fasting glucose is more prevalent in men, and impaired glucose tolerance is more prevalent in women (Mauvais-Jarvis, 2018[87]). However, a survey conducted in 2009 in the field of endocrinology reports that about 11.2 % of animal research was done in both sexes, only 20.7 % in females, and 66 % in males (2.1 % unsuccessful in specifying the sex of experimental models), indicating a male bias of 3.2:1 (Beery and Zucker, 2011[8]). This sex bias causes a generalization of findings in men to women without appropriate justification (Beery and Zucker, 2011[8]). Surprisingly, some papers suggest that researchers prefer male animals over females to diminish mortality and increase the efficacy of STZ (Goyal et al., 2016[41]). This recommendation is unlike NIH policy to avoid over-reliance on male-only animal models and to balance both sexes in the preclinical studies (Clayton and Collins, 2014[16]) that have been further discussed elsewhere (McCullough et al., 2014[88]). Interested readers are referred to an excellent review by Ritz et al. for addressing sex and gender considerations in basic biomedical research (Ritz et al., 2014[107]). 

Different sensitivity to STZ-induced diabetes has been reported in male and female mice. In CD-1 mice, following STZ injection (40 mg/kg, IP) for 5 days, plasma glucose concentrations, measured five days after the last dose of STZ, were significantly higher in males than females by about 75 % (356±18 vs. 205±9 mg/dL) (Rossini et al., 1978[113]); in addition, plasma glucose concentrations were similar between castrated males and control females and between testosterone-administrated female and control males (Rossini et al., 1978[113]). Castrated females had higher glucose than control females, whereas castrated males had lower plasma glucose than control males (Rossini et al., 1978[113]). In another study, in C57BL/6J mice, following STZ (40 mg/kg, IP) injection for 5 days, fasting blood glucose was about 50 % higher in males than females (236±26 vs. 158±17 mg/dL) at 5 weeks after STZ injection (Kim et al., 2020[67]). Another study in C57BL/6 mice reported similar results 8 weeks after STZ (55 mg/kg, IP, for 5 consecutive days) injection, i.e., blood glucose was about 90 % higher in males than females (462±23 vs. 239±31 mg/dL). These data indicate that male rodents are more sensitive to STZ and tend to develop greater hyperglycemia, as mentioned previously (Tesch and Allen, 2007[133]). The resistance of female mice to the diabetogenic effect of STZ can be overcome by increasing the amount of injected STZ; this issue has been reported in C57BL/6J mice, where a higher dose of STZ in females (75 mg/kg, IP for 5 consecutive days) than males (55 mg/kg, IP for 5 consecutive days) achieved same levels of hyperglycemia (Saadane et al., 2020[114]).

## Age-Dependent Susceptibility to STZ

The mass of β-cells in both humans (Butler et al., 2003[11]) and rodents (Finegood et al., 1995[24]) is dynamic, and the life span of rat β-cells is 1-3 months (Finegood et al., 1995[24]). Rat β-cell mass increases sharply after birth (with a plateau in post-natal days of 5-20) until about post-natal day 100 and has a slower growth after that (Finegood et al., 1995[24]). Mass of β-cells is regulated by the balance in β-cell growth, i.e., changes in cell size/volume (hypertrophy vs. hypotrophy) and β-cell number (cell production by neogenesis and replication vs. cell loss by apoptosis and necrosis) (Finegood et al., 1995[24]; Masiello, 2006[82]). β-cell volume is 510 μm^3^ in rats younger than 21 days, reaches 1020 μm^3^ at about post-natal day 21, and remains constant after that, at least until 500 days (Finegood et al., 1995[24]). New pancreatic β-cells are formed by neogenesis (differentiation from undifferentiated precursors such as embryonic duct cells) mostly during gestation and also by replication (formation of new cells from preexisting differentiated cells) mostly after birth (Finegood et al., 1995[24]). In rats older than 30-40 days, the rate of β-cells duplication is about 3 % per day and, along with hypertrophy of β-cells (Finegood et al., 1995[24]), plays a significant role in the regulation of β-cell mass (Finegood et al., 1995[24];[24] Butler et al., 2003[11]) with little contribution of neogenesis (Finegood et al., 1995[24]). β-cell replication in rats is age-dependent (Finegood et al., 1995[24]); it is highest at birth, reaches about 3 % by post-natal days 30-40, and remains constant after that (Finegood et al., 1995[24]). β-cell replication in rats can be estimated by the equation provided by Finegood et al. (e=2.718) (Finegood et al., 1995[24]): 


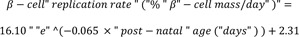


Unlike rats, replication and changes in β-cell size have little importance in humans; in humans with type 2 diabetes, the neogenesis rate is constant, and increased β-cell apoptosis is primarily involved in regulating β-cell mass (Butler et al., 2003[11]). 

Susceptibility to the diabetogenic effect of STZ is inversely related to animal age; in male Wistar rats aged 25-50 days, higher doses of STZ are needed to induce diabetes successfully in younger rats (Masiello et al., 1975[84], 1979[85]). For example, the dose of STZ needed to make diabetes in 50 g rats (about 25 days of age) is twice higher than that required for 150 g rats (about 50 days of age) (Masiello et al., 1975[84]). Age-dependent susceptibility to STZ may be related to rats' decreased capacity for β-cell regeneration (Swenne, 1983[128]). This notion is supported by findings that doses of STZ required for inducing diabetes in neonatal rats (80-100 mg/kg) are about 30-40 % higher than doses in adult rats (60 mg/kg); in addition, neonatal STZ-induced diabetes in rats is characterized by rapid and spontaneous recovery (Portha et al., 1974[101]). In neonatal STZ rats, the β-cells number per pancreas is about 50 % lower in n5-STZ compared to n0-STZ rats, as measured 3 weeks later (0.44±0.04 × 10^6 ^vs. 0.84±0.07× 10^6^) (Wang et al., 1996[137]). Age-dependent reduction in insulin secretion and elevation in blood glucose has also been reported in male Wistar rats; in the presence of glucose concentration of 5 mM, the amount of insulin secreted from a single β-cell isolated from 24-months-old rats was 34 % lower than 6-months-old rats (Perfetti et al., 1995[99]). 

## Strain-Dependent Susceptibility to STZ

More than 1000 rat strains have been used for research (Reed et al., 2011[104]). Strain difference in the susceptibility to STZ has been reported in mice (Kaku et al., 1989[61]; Hayashi et al., 2006[45]) and rats (Rodrigues et al., 1997[110]). Male Wistar-Kyoto rats are less sensitive to a moderate (55 mg/kg, IV) dose of STZ than Wistar rats, as demonstrated with a lesser degree of non-fasted hyperglycemia (414±41 vs. 533±13 mg/dL) (Rodrigues et al., 1997[110]). This can be overcome by administrating a greater dose of STZ (75 mg/kg), which provided comparable hyperglycemia between these two strains of rats (Rodrigues et al., 1997[110]). In addition, it has been shown that, unlike spontaneously hypertensive rats, male Wistar-Kyoto rats are resistant to the n2-STZ-induced diabetic model (Iwase et al., 1987[55]). Dark Agouti (DA) and Albino Oxford (AO) rats are susceptible to single high-dose (SHD)-STZ-induced diabetes, but DA rats are highly vulnerable, and AO are resistant to multiple low-dose (MLD)-STZ-induced diabetes (Lukić et al., 1998[76]; Howarth et al., 2005[48]). Wistar and Sprague-Dawley rats, the most commonly-used rat strains for STZ-induced diabetes, are sensitive to STZ (Samuel et al., 2014[115]).

## Appropriate Route of STZ Administration

STZ is mostly injected through IV and IP routes; however, intracardiac, subcutaneous, and intramuscular administration of STZ have also been reported (Deeds et al., 2011[19]; Ghasemi et al., 2014[36]). Intraperitoneal injection of STZ is easier, much more used, and has a high reproducibility (Chaudhry et al., 2013[14]; Gvazava et al., 2020[44]). However, it has been found to be associated with risk of intestinal injury and mortality, and its penetration into the subcutaneous tissue reduces its diabetogenic effect (Deeds et al., 2011[19]; Gvazava et al., 2020[44]). Thus STZ dosage required to achieve the same level of diabetes via the IP route is higher than the IV route (Tesch and Allen, 2007[133]). It has been reported that IV injection of STZ to mice produces a more reproducible and stable diabetic model (Tay et al., 2005[131]).

## Rat Mortality after STZ Injection

Following the injection of diabetogenic doses (e.g., 65 mg/kg) of STZ, mortality is due to severe hypoglycemia during the first 24 h (Junod et al., 1967[58]; Fischer and Rickert, 1975[25]; Gajdosík et al., 1999[30]) or severe hyperglycemia that occurred after that (Junod et al., 1969[59]). It has been reported that days 2 and 3 after STZ injection is the most critical period regarding the mortality rate (Gajdosík et al., 1999[30]). In a study in male Wistar rats, mortality rates were 1 out of 8, 2 out of 8, and 8 out of 8 during 2-3 days following 50, 60, and 70 mg/kg STZ injection (IV) to overnight-fasted animals (Gajdosík et al., 1999[30]). Following STZ (65 mg/kg, IV) injection to fasted (24 h) male Sprague-Dawley rats, the six-week mortality rate was 12 % (3 out of 25) (Hoftiezer and Carpenter, 1973[46]).

Measures to prevent early hypoglycemia-related mortality following STZ injection in rats include providing access to food soon after the injection (Junod et al., 1967[58], 1969[59]), administration of glucose (Fischer and Rickert, 1975[25]; Bar-On et al., 1976[6]; Babu and Srinivasan, 1997[4]; Palsamy and Subramanian, 2011[97]) or sucrose (Tesch and Allen, 2007[133]; Ramzy et al., 2014[103]) solutions in the first 24-48 h after the injection, administration of the drug to fed animals (Gajdosík et al., 1999[30]), and using the anomer-equilibrated solutions of STZ (de la Garza-Rodea et al., 2010[18]).

Early hypoglycemia-related mortality is more pronounced in fasted animals (Gajdosík et al., 1999[30]) and can be prevented by providing rat access to food soon after injection (Junod et al., 1967[58], 1969[59]). Administration of glucose (10 % from 6-24 h after STZ injection (Palsamy and Subramanian, 2011[97]) and 5 % during the first 24 h after STZ injection (Bar-On et al., 1976[6]; Babu and Srinivasan, 1997[4])) and sucrose (10 % during 48 h after STZ injection (Ramzy et al., 2014[103]) and 1.5 % during 48 h after STZ injection (Tesch and Allen, 2007[133])) solutions have been used to prevent hypoglycemia-related early mortality in rats. 

Administration of the STZ to the fed animals can also decrease hypoglycemia-related mortality (Gajdosík et al., 1999[30]). Following the injection of high doses (100 mg/kg) of STZ to fasted rats, most rats died within 2-3 days (Junod et al., 1969[59]). Following STZ (50 mg/kg, IP) injection to fasted (16 h) rats of both sexes, 3 out of 6 rats died during 14 days (Mythili et al., 2004[92]). On the other hand, after injection of STZ (100 mg/kg, IV) to non-fasted rats, the mortality rate was 12.5 % after 1 and 4 weeks and increased to 29 % after 16 weeks (Tancrède et al., 1983[129]). No mortality was reported when STZ was injected to non-fasted male Wistar rats treated with doses ≤ 65 mg/kg IV within 16 weeks (Tancrède et al., 1983[129]) or ≤ 45 mg/kg IV to non-fasted male Sprague-Dawley rats within 12 days after injection (Bar-On et al., 1976[6]). Only, one out of six non-fasted male Sprague-Dawley rats died following the IV injection of 55 mg/kg STZ within 12 days (Bar-On et al., 1976[6]).

Hyperglycemia-related mortality following injection of high doses of STZ can be overcome with insulin administration (Junod et al., 1969[59]). Rodent by non-fasting blood glucose between 290-540 mg/dL can be maintained without insulin injection, and those with non-fasting blood glucose higher than 630 mg/dL needs insulin therapy (Tesch and Allen, 2007[133]). The dose of insulin required varies according to the severity of the disease, species, and strain and should be determined by the researcher (Tesch and Allen, 2007[133]) but is around 10-15 U/kg/day (Junod et al., 1969[59]) or 2-4 U/rat/day (Tesch and Allen, 2007[133]) of subcutaneous injections of long-acting insulin.

## Conclusion

**Figure 3:**
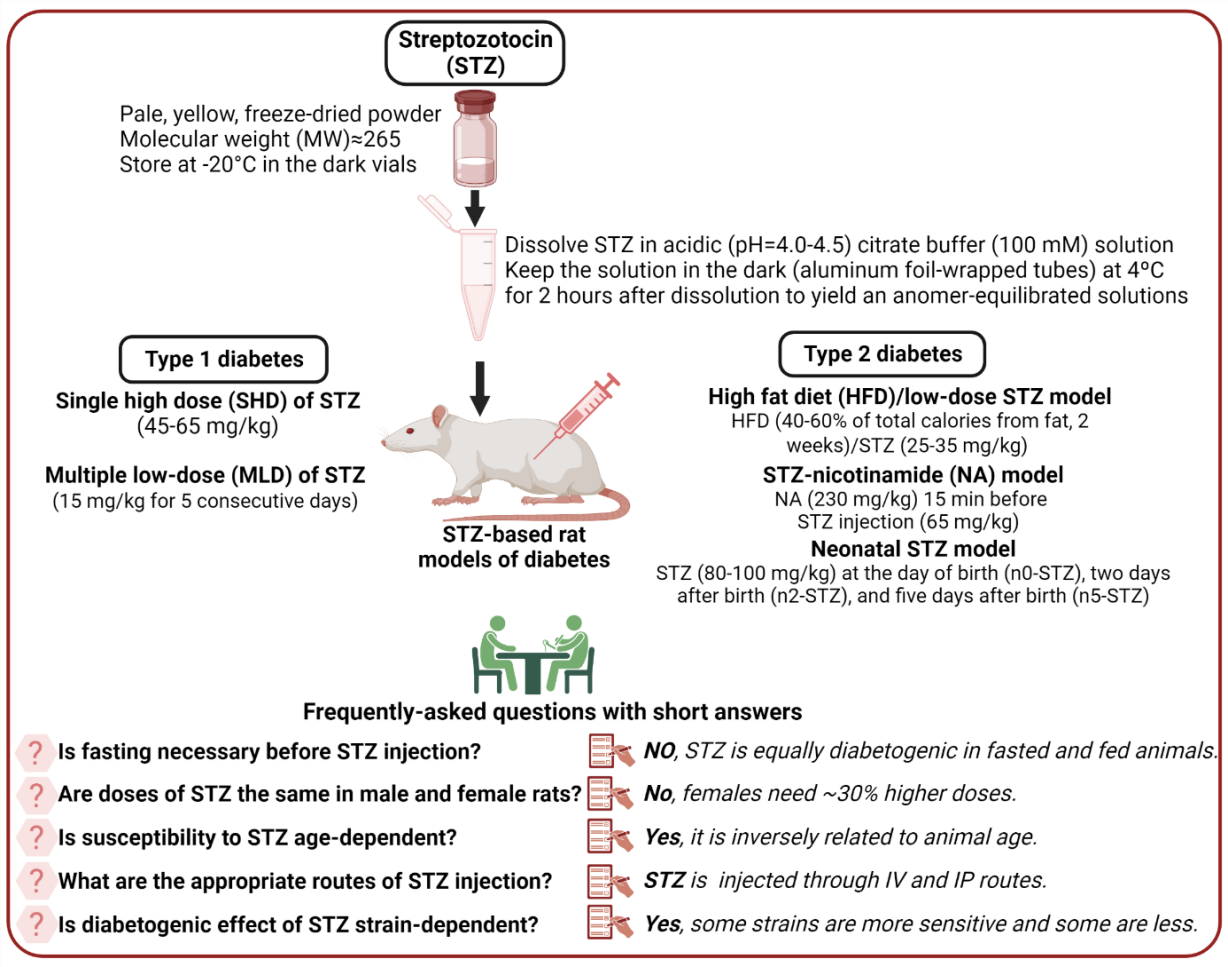
A practical guide for maintaining, preparing, and injecting streptozotocin (STZ) in STZ-based rat models of diabetes. IV, intravenous; IP, intraperitoneal. Created with BioRender.com

Based on the presented discussion, a practical guide is provided in Figure 3(Fig. 3) that can help diabetes researchers to conduct better studies. In addition, some points deserve further attention and are briefly presented here. First, in addition to the intrinsic limitations of animal models, poor design and reporting of animal studies are the main causes of poor concordance between preclinical and clinical outcomes (Perrin, 2014[100]; Bahadoran et al., 2020[5]). Further attention should be paid to reporting the results of animal studies. Despite available guidelines on reporting results of animal studies (Hooijmans et al., 2010[47]; Osborne et al., 2018[96]; Percie du Sert et al., 2020[98]; Nagendrababu et al., 2021[93]), a high number of papers fail appropriately report; for example, they do not provide animal age or sex (Kilkenny et al., 2009[66]; Flórez-Vargas et al., 2016[26]), which can lead to a reproducibility crisis in biomedical research (Osborne et al., 2018[96]). Thus, it is recommended to report details in the method section, including animal age and sex, preparation of STZ, exact time of fasting before blood sampling, the success rate of inducing diabetes, and mortality rate (de la Garza-Rodea et al., 2010[18]; Ghasemi et al., 2021[35]). Second, considering the large body of evidence indicating sex differences in carbohydrate metabolism (Tarnopolsky and Ruby, 2001[130]; Basu et al., 2006[7]; Wismann and Willoughby, 2006[140]; Franconi et al., 2008[27]; Macotela et al., 2009[78]; Gustavsson et al., 2010[43], 2011[42]; Marchese et al., 2015[81]; Gannon et al., 2018[32]; Mauvais-Jarvis, 2018[87]) and in STZ sensitivity in animals (Rossini et al., 1978[113]; Kim et al., 2020[67]; Saadane et al., 2020[114]), over-reliance on male-only animal models should be avoided, and researchers should try to balance both sexes in the preclinical studies (Clayton and Collins, 2014[16]; McCullough et al., 2014[88]; Ritz et al., 2014[107]). Even most initial studies that developed rat models of diabetes used only male rats (see Table 1(Tab. 1)), a practice that needs revision. Third, diabetes researchers need a better understanding of animal models of diabetes -this issue helps to obtain robust results that have more chance to be translated to humans. In addition, it helps to adhere to ethical issues in animal research, for example, by choosing the optimal dose of STZ that reduces the mortality rate and the number of animals used. Finally, differences between rats and humans should be considered in animal modeling, including differences in gene regulation, different life spans, and different metabolic rates (Wall and Shani, 2008[136]). For choosing an appropriate animal model for diabetes research, the purpose of the study, animal sex, animal strain, and physiological relevance of the model used should be considered (King, 2012[68]).

## Declaration

### Acknowledgments and funding information

This study was supported by a grant (Grant No. 43004018-7) from Shahid Beheshti University of Medical Sciences, Tehran, Iran.

### Declaration of competing interest 

The authors declare that they have no competing interests.

### Authorships

Asghar Ghasemi contributed to the literature review and wrote the article. Sajad Jeddi and Asghar Ghasemi provided critical revision and final approval of the finalized manuscript. All authors have read and approved the final manuscript.

## Figures and Tables

**Table 1:**
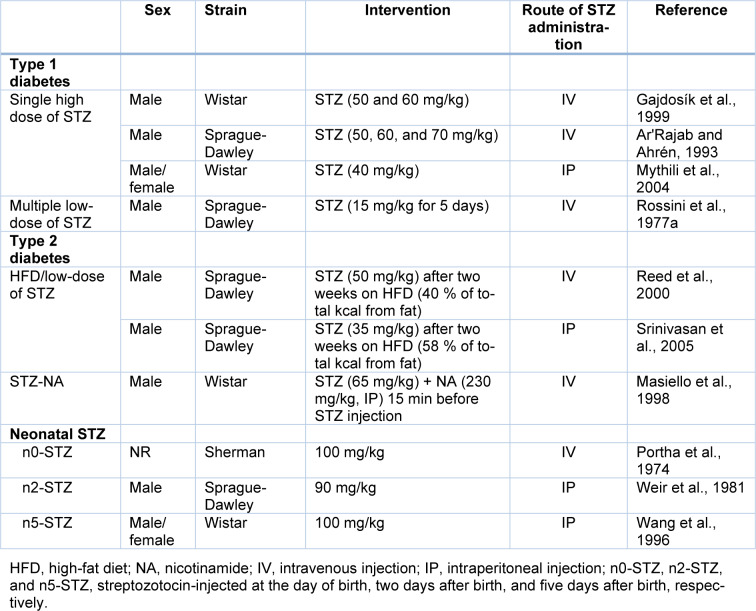
Rat models of streptozotocin (STZ) diabetes
